# “AI-powered triage: the missing link in Pakistan’s telemedicine rollout”

**DOI:** 10.1097/MS9.0000000000005087

**Published:** 2026-04-29

**Authors:** Tooba Adil, Esha Khan, Muhammad Umer Suleman, Syeda Nafisa Tabassum

**Affiliations:** a Department of Community Medicine, Jinnah Sindh Medical University, Pakistan; b Nishtar Medical University, Multan, Pakistan; c Department of Internal Medicine, Ayub Medical College, Abbottabad, Pakistan; d Department of Microbiology, Bangladesh Rural Advancement Committee (BRAC), Dhaka, Bangladesh

*Dear Editor,*

Pakistan is moving toward a historic shift in healthcare delivery, as telemedicine centers are expected to become functional nationwide by June 2026[1]. Federal leadership has repeatedly emphasized that nearly 70% of patients bypass BHUs and head directly to tertiary hospitals, creating unnecessary overcrowding that telemedicine aims to alleviate[1]. However, without artificial intelligence (AI)-powered triage, telemedicine will remain an access tool rather than an effective clinical system capable of transforming primary healthcare.

Provincial and federal initiatives reflect strong momentum. The CDA plans to introduce telemedicine across all BHUs and RHCs in Islamabad to expand access and reduce pressure on PIMS, Polyclinic, and CDA Hospital[2]. National telemedicine centers under the Ministry of Health are also targeted for rollout by mid-2026[3]. Existing successful models show the potential impact: ChildLife Foundation’s telemedicine network, recently recognized at the National Health Awards, connects over 300 hospitals with remote pediatric specialists, demonstrating improved outcomes in emergency care[4]. Similarly, Khyber Pakhtunkhwa has established pediatric telemedicine centers to allow real-time expert guidance via video link, significantly reducing avoidable delays[5].

However, the Wafaqi Mohtasib Secretariat Report on Islamabad’s PHC system highlights why telemedicine needs more than video consultations alone. The report reveals that 55 PHC facilities collectively see only 12–13 patients per day despite being staffed with 579 workers, with some centers lacking doctors entirely and others suffering severe staffing imbalances[6]. The referral system is described as rudimentary and poorly coordinated, leading to ineffective patient flow. Telemedicine, although acknowledged in policy, remains non-functional in rural PHCs despite its known benefits for Pakistan’s largely rural population[7]. The report recommends immediate introduction and strengthening of telemedicine as a priority[8].

Additional evidence from the *Cureus Scoping Review* on AI triage reinforces the value of integrating intelligent systems into emerging telemedicine platforms. The review found that AI and machine-learning models consistently outperform traditional triage systems, providing higher accuracy, reducing under-triage and over-triage, improving prediction of hospital admissions, and detecting clinical deterioration earlier[9]. AI-enhanced triage tools were shown to streamline emergency workflows, optimize resource use, and support rapid, evidence-based decision-making, benefits directly applicable to Pakistan’s underperforming PHC system[9].

As Pakistan prepares for national telemedicine deployment in June 2026, integrating AI-powered triage into BHU and RHC digital clinics is essential. Without AI-driven urgency detection and standardized assessment, telemedicine may expand access but fail to improve outcomes. With AI, however, telemedicine can become a smarter, safer, and more impactful public health intervention.

Our work is in line with the TITAN Guidelines on the need for transparency in AI use in healthcare[10].

## Data Availability

Data sharing does not apply to this article as no datasets were generated during the current study; all data were sourced from published literature.
